# Revealing Hidden Diversity of the Underestimated Neotropical Ichthyofauna: DNA Barcoding in the Recently Described Genus *Megaleporinus* (Characiformes: Anostomidae)

**DOI:** 10.3389/fgene.2017.00149

**Published:** 2017-10-12

**Authors:** Jorge L. Ramirez, Jose L. Birindelli, Daniel C. Carvalho, Paulo R. A. M. Affonso, Paulo C. Venere, Hernán Ortega, Mauricio Carrillo-Avila, José A. Rodríguez-Pulido, Pedro M. Galetti

**Affiliations:** ^1^Laboratório de Biodiversidade Molecular e Conservação, Departamento de Genética e Evolução, Universidade Federal de São Carlos, São Paulo, Brazil; ^2^Departamento de Biologia Animal e Vegetal, Universidade Estadual de Londrina, Londrina, Brazil; ^3^Laboratório de Genética da Conservação, Programa de Pós-Graduação em Biologia de Vertebrados, PUC Minas, Belo Horizonte, Brazil; ^4^Departamento de Ciências Biológicas, Universidade Estadual do Sudoeste da Bahia, Jequié, Brazil; ^5^Departamento de Biologia e Zoologia, Universidade Federal de Mato Grosso, Cuiabá, Brazil; ^6^Departamento de Ictiología, Museo de Historia Natural, Universidad Nacional Mayor de San Marcos, Lima, Peru; ^7^Facultad de Ciencias Exactas y Naturales, Universidad Surcolombiana, Huila, Colombia; ^8^Grupo de Investigación en Genética y Reproducción Animal, Universidad de los Llanos, Villavicencio, Colombia

**Keywords:** cryptic species, freshwater fishes, allopatric speciation, South American basins, cytochrome oxidase subunit I

## Abstract

Molecular studies have improved our knowledge on the neotropical ichthyofauna. DNA barcoding has successfully been used in fish species identification and in detecting cryptic diversity. *Megaleporinus* (Anostomidae) is a recently described freshwater fish genus within which taxonomic uncertainties remain. Here we assessed all nominal species of this genus using a DNA barcode approach (Cytochrome Oxidase subunit I) with a broad sampling to generate a reference library, characterize new molecular lineages, and test the hypothesis that some of the nominal species represent species complexes. The analyses identified 16 (ABGD and BIN) to 18 (ABGD, GMYC, and PTP) different molecular operational taxonomic units (MOTUs) within the 10 studied nominal species, indicating cryptic biodiversity and potential candidate species. Only *Megaleporinus brinco, Megaleporinus garmani*, and *Megaleporinus elongatus* showed correspondence between nominal species and MOTUs. Within six nominal species, a subdivision in two MOTUs was found, while *Megaleporinus obtusidens* was divided in three MOTUs, suggesting that DNA barcode is a very useful approach to identify the molecular lineages of *Megaleporinus*, even in the case of recent divergence (< 0.5 Ma). Our results thus provided molecular findings that can be used along with morphological traits to better define each species, including candidate new species. This is the most complete analysis of DNA barcode in this recently described genus, and considering its economic value, a precise species identification is quite desirable and fundamental for conservation of the whole biodiversity of this fish.

## Introduction

Neotropical freshwater fishes have a remarkable diversity, exceeding 8000 species (Reis et al., 2016), however, much taxonomic uncertainty exists leading to underestimated diversity (Pereira et al., 2013; Reis et al., 2016). Molecular studies have been crucial to improve our knowledge on the ichthyofauna, and DNA barcoding has successfully been used in fish species identification and in detecting species of taxonomic concerns or cryptic diversity (Pereira et al., 2013; Gomes et al., 2015; Ramirez and Galetti, 2015; Machado et al., 2016). Within the neotropical freshwater fishes, the order Characiformes represents more than 30% of the known species, and Anostomidae is one of the most species-rich families, occurring in all major hydrographic basins, with *trans*- and *cis*-Andean distribution in South America (Reis et al., 2003).

Comprising approximately 150 described species, distributed in 15 genera (Garavello and Britski, 2003; Sidlauskas and Vari, 2008; Ramirez et al., 2017), the known diversity of the Anostomidae has increased in recent years. For instance, 14 species and 1 genus were described only in the last 5 years (Birindelli et al., 2013; Burns et al., 2014). DNA barcoding has revealed taxonomic uncertainties within the genus *Laemolyta* (Ramirez and Galetti, 2015), and molecular phylogeny has helped to provide an understanding of the evolutionary history of the Anostomidae (Ramirez and Galetti, 2015; Ramirez et al., 2016, 2017).

Recently, the genus *Megaleporinus* (Ramirez et al., 2017) was described to include 16 lineages, corresponding to 10 nominal species, previously recognized in *Leporinus* or *Hypomasticus* (Ramirez et al., 2017). *Megaleporinus* is supported by cytogenetic, molecular, and morphological data. It is characterized by having a unique ZZ/ZW sex chromosome system (Galetti et al., 1995), while most cytogenetically known *Leporinus* species have no sex chromosomes (Galetti et al., 1981, 1991). Its monophyly is also well supported by mitochondrial and nuclear markers, which identified it as the sister group to *Abramites* (Ramirez et al., 2017). Concerning its morphology, *Megaleporinus* is characterized by being relatively large (adults usually reaching more than 35 cm standard length, including the largest species of the family), three teeth on each premaxillary and dentary bones, and a color pattern of one to three dark mid-lateral blotches (Ramirez et al., 2017). Because of its large size, *Megaleporinus* has an economic importance in subsistence fisheries and aquaculture (Garavello and Britski, 2003).

Recent studies indicate that there is a hidden biodiversity within *Megaleporinus* that needs to be better understood (Avelino et al., 2015; Ramirez et al., 2017). A study based on mitochondrial and nuclear markers, but using few individuals for each species, showed that several nominal species allocated to this genus comprise two or more molecular lineages allopatrically distributed in different basins (Ramirez et al., 2017).

In this study, we used a DNA barcoding approach to generate a reference library for *Megaleporinus*, assessing all nominal species and lineages previously described. We included a broad sampling for most of the species. Our hypothesis is that DNA barcoding support the observation that some of the nominal species represent species complexes with most molecular operational taxonomic units (MOTUs) allopatrically distributed in different basins, as proposed by Ramirez et al. (2017). Identifying such hidden biodiversity within this genus, this paper will contribute to a more complete understanding of its diversity and to the conservation of this important fish group.

## Materials and Methods

### Sampling

Animals were collected on public land, handled and killed under permission (ICMBIO/MMA N° 32215) provided by the Environment Ministry (MMA). This study did not involve endangered or protected species. Fish were collected by fishing rods and gillnets. No ethics committee approval is required for these organisms in Brazil. Fish were killed in the field using cold water and immediately transferred onto ice. Tissue samples were collected after fish death was confirmed through lack of operculum movement.

**FIGURE 1:**
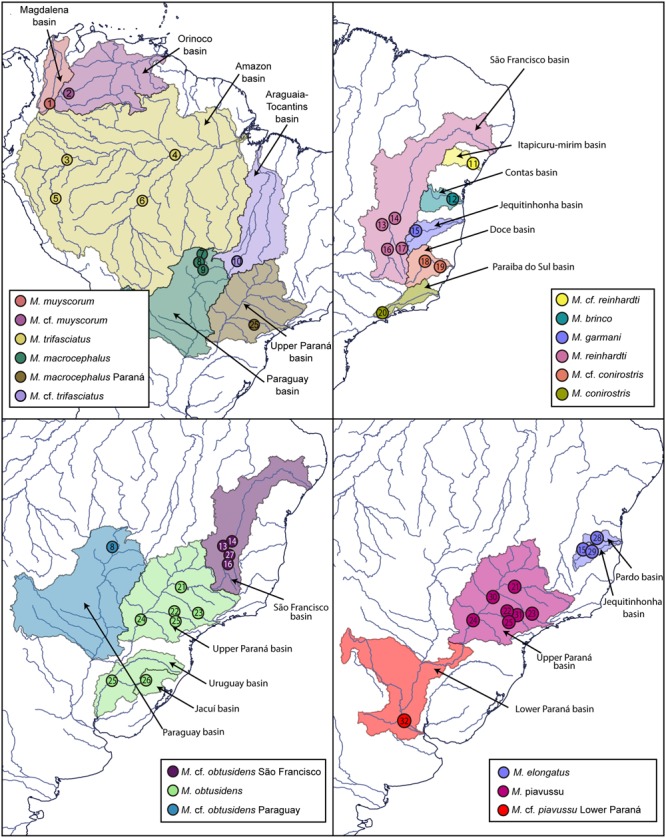
Collection sites (circles) and hydrographic basin of occurrence of *Megaleporinus* MOTUs. Localities’ numbers according to **Table 1**.

**FIGURE 2:**
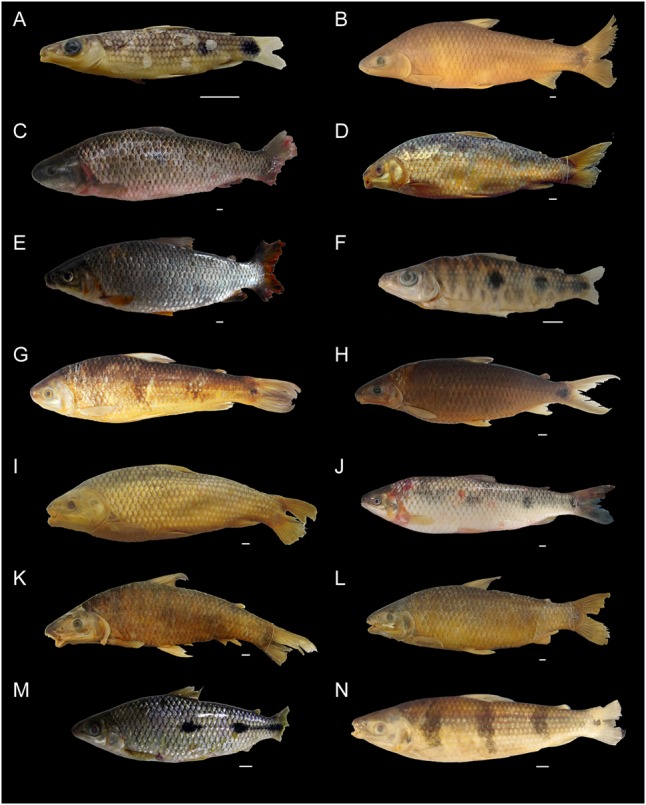
Studied specimens of *Megaleporinus*. **(A)** *M. brinco*, MZUSP 118670; **(B)** *M.* cf. *conirostris*, LISDEBE 6971; **(C)** *M.* cf. *muyscorum*; **(D)** *M*. cf. *obtusidens* Paraguay, MZUSP 118668; **(E)** *M.* cf. *obtusidens* São Francisco, MCP 44805; **(F)** *M*. cf. *reinhardti*, UESB-8206; **(G)** *M*. cf. *trifasciatus*, GEPEMA 5095; **(H)** *M. garmani*, MCNI-PUCMG-0020; **(I)** *M. macrocephalus*, MZUSP 118667; **(J)** *M. muyscorum*, ICN-19074; **(K)** *M. obtusidens*, MZUSP 113982; **(L)** *M. piavussu*, MZUSP 113981; **(M)** *M. reinhardti*; **(N)** *M. trifasciatus*, MUSM – 47351. Scale bars equal 1 cm.

Specimens from several populations of all *Megaleporinus* species were used in this study, totaling 79 samples of the 10 nominal species, and comprising the 16 molecular lineages described by Ramirez et al., 2017 (**Figures 1, 2** and **Table 1**). Voucher numbers are provided for the specimens (**Table 1**). Additionally, previous DNA barcode sequences of specimens from the São Francisco (Carvalho et al., 2011), Paraná (Pereira et al., 2013), Paranapanema (Frantine-Silva et al., 2015), and lower Paraná basins (Díaz et al., 2016) were included in our data set giving a total of 116 sequences (**Figures 1, 2** and **Table 1**).

**Table 1:** Sampling information and GenBank accession for specimens included in the analysis.

MOTU	River (Locality)	Basin	BIN	GenBank	Museum ID
*Megaleporinus brinco*	Contas^12^	Contas	ADB0463	KU134850	MZUSP – 118670
*M. brinco*	Contas^12^	Contas	ADB0463	KX925449	MZUSP – 118670
*M. brinco*	Contas^12^	Contas	ADB0463	KX925450	MZUSP – 118670
*M.* cf. *conirostris*	Doce (Governador Valadares)^18^	Doce	ACL4264	KF568977	MCNI-PUCMG-0186
*M.* cf. *conirostris*	Doce (Governador Valadares)^18^	Doce	ACL4264	KX925451	–
*M.* cf. *conirostris*	Doce (Governador Valadares)^18^	Doce	ACL4264	KX925452	–
*M.* cf. *conirostris*	Doce (Baixo Guandú)^19^	Doce	ACL4264	KX925453	LISDEBE 6971
*M.* cf. *muyscorum*	Meta (Puerto Lopez)^2^	Orinoco	ADB0512	KU134851	–
*M.* cf. *obtusidens* Paraguay	Cuiaba (Santo Antonio de Leverger)^8^	Paraguai	ACL3942	KU134861	MZUSP – 118668
*M.* cf. *obtusidens* São Francisco	São Francisco (Tres Marias)^20^	São Francisco	ABZ0928	HM405029	^∗^
*M.* cf. *obtusidens* São Francisco	Pandeiros^14^	São Francisco	ABZ0928	HM405142	^∗^
*M.* cf. *obtusidens* São Francisco	Urucuia (Urucuia)^13^	São Francisco	ABZ0928	HM906022	^∗^
*M.* cf. *obtusidens* São Francisco	Urucuia (Urucuia)^13^	São Francisco	ABZ0928	HM906023	^∗^
*M.* cf. *obtusidens* São Francisco	São Francisco (Tres Marias)^16^	São Francisco	ABZ0928	HM405028	^∗^
*M.* cf. *obtusidens* São Francisco	São Francisco (Pirapora)^27^	São Francisco	ABZ0928	KX925498	LISDEBE 6973
*M.* cf. *obtusidens* São Francisco	São Francisco (Pirapora)^27^	São Francisco	ABZ0928	KX925499	–
*M.* cf. *obtusidens* São Francisco	São Francisco (Pirapora)^27^	São Francisco	ABZ0928	KX925500	–
*M.* cf. *obtusidens* São Francisco	São Francisco (Pirapora)^27^	São Francisco	ABZ0928	KX925501	–
*M.* cf. *obtusidens* São Francisco	São Francisco (Pirapora)^27^	São Francisco	ABZ0928	KX925502	–
*M.* cf. *obtusidens* São Francisco	Pandeiros (Pandeiros)^14^	São Francisco	ABZ0928	KU134862	MCP – 44805
*M.* cf. *obtusidens* São Francisco	Urucuia (Urucuia)^13^	São Francisco	ABZ0928	KX925503	–
*M.* cf. *obtusidens* São Francisco	Urucuia (Urucuia)^13^	São Francisco	ABZ0928	KX925504	MCP – 44076
*M.* cf. *reinhardti*	Itapicurú^11^	Itapicurú-mirim	AAD1729	KU134849	–
*M.* cf. *reinhardti*	Itapicurú^11^	Itapicurú-mirim	AAD1729	KX925454	–
*M.* cf. *reinhardti*	Itapicurú^11^	Itapicurú-mirim	AAD1729	KX925455	–
*M.* cf. *reinhardti*	Itapicurú^11^	Itapicurú-mirim	AAD1729	KX925456	UESB-8206
*M.* cf. *trifasciatus*	Araguaia (Ouro fino)^10^	Tocantins	ACL3074	KX925457	GEPEMA – 4975
*M.* cf. *trifasciatus*	Araguaia (Barra do Garças)^10^	Tocantins	ACL3074	KF568998	GEPEMA – 5095
*M.* cf. *trifasciatus*	Araguaia (Barra do Garças)^10^	Tocantins	ACL3074	KX925458	GEPEMA – 5594
*M. conirostris*	Paraibuna^20^	Paraiba do Sul	ACL3731	KU134852	–
*M. conirostris*	Paraibuna^20^	Paraiba do Sul	ACL3731	KX925459	–
*M. elongatus*	Itacambiruçu (Grão Mogol)^15^	Jequitinhonha	ABY2894	KX925463	–
*M. elongatus*	Itacambiruçu (Grão Mogol)^15^	Jequitinhonha	ABY2894	KU134853	MCNI-PUCMG-0375
*M. elongatus*		Jequitinhonha	ABY2894	KX925464	MCNI-PUCMG-0221
*M. elongatus*	Jequitinhonha (UHE Irapé)^29^	Jequitinhonha	ABY2894	KU134854	–
*M. elongatus*	Jequitinhonha (UHE Irapé)^29^	Jequitinhonha	ABY2894	KX925465	MCNI-PUCMG-0299
*M. elongatus*	Jequitinhonha (UHE Irapé)^29^	Jequitinhonha	ABY2894	KX925466	MCNI-PUCMG-0300
*M. elongatus*	Rio Pardo (Águas Vermelhas)^28^	Pardo	ABY2894	KX925460	MCNI-PUCMG-4451
*M. elongatus*	Rio Pardo (Águas vermelhas)^28^	Pardo	ABY2894	KX925461	MCNI-PUCMG-5175
*M. elongatus*	Rio Pardo (Águas vermelhas)^28^	Pardo	ABY2894	KX925462	MCNI-PUCMG-5176
*M. garmani*	Itacambiruçu (Grão Mogol)^15^	Jequitinhonha	ACL3227	KU134855	MCNI-PUCMG-0021
*M. garmani*	Itacambiruçu (Grão Mogol)^15^	Jequitinhonha	ACL3227	KX925467	MCNI-PUCMG-0020
*M. garmani*	Itacambiruçu (Grão Mogol)^15^	Jequitinhonha	ACL3227	KX925468	MCNI-PUCMG-0021
*M. garmani*	Itacambiruçu (Grão Mogol)^15^	Jequitinhonha	ACL3227	KX925469	MCNI-PUCMG-0021
*M. garmani*	Itacambiruçu (Grão Mogol)^15^	Jequitinhonha	ACL3227	KX925470	MCNI-PUCMG-0374
*M. macrocephalus*	Manhuaçu (São José do Mantimento)	Doce	AAE5328	KX925475	MCNI-PUCMG-0460
*M. macrocephalus*	Cuiaba (Santo Antonio de Leverger)^8^	Paraguai	AAE5328	KU134856	MZUSP – 118667
*M. macrocephalus*	Cuiaba (Santo Antonio de Leverger)^8^	Paraguai	AAE5328	KX925471	LISDEBE 6972
*M. macrocephalus*	Cuiaba (Cuiaba)^7^	Paraguai	AAE5328	KX925474	–
*M. macrocephalus*	Cuiaba (Barão de Melgaço)^9^	Paraguai	AAE5328	KX925476	LISDEBE 6974
*M. macrocephalus*	Cuiaba (Barão de Melgaço)^9^	Paraguai	AAE5328	KX925477	LISDEBE 6974
*M. macrocephalus*	Tiete (Barra Bonita)	Paraná	AAE5328	KX925473	–
*M. macrocephalus*	Pandeiros	São Francisco	AAE5328	HM906021	–
*M. macrocephalus*	Araguaia (Ouro fino)	Tocantins	AAE5328	KX925472	GEPEMA – 4974
*M. macrocephalus* Paraná	Cinzas (Bandeirantes)^25^	Paraná	ACO1303	KM897611	^∗^
*M. macrocephalus* Paraná	Cinzas (Bandeirantes)^25^	Paraná	ACO1303	KM897537	^∗^
*M. macrocephalus* Paraná	Cinzas (Bandeirantes)^25^	Paraná	ACO1303	KM897575	^∗^
*M. macrocephalus* Paraná	Cinzas (Bandeirantes)^25^	Paraná	ACO1303	KM897296	^∗^
*M. macrocephalus* Paraná	Piracicaba (Tamanduá)^23^	Paraná	ACO1303	JN988999	LBPV-19469
*M. muyscorum*	Magdalena (Neiva)^1^	Magdalena	ADB0701	KX925478	ICN-19072
*M. muyscorum*	Magdalena (Neiva)^1^	Magdalena	ADB0701	KX925479	ICN-19073
*M. muyscorum*	Magdalena (Neiva)^1^	Magdalena	ADB0701	KU134857	ICN-19074
*M. obtusidens*	Jacuí (Jacuizinho Foz)^26^	Jacuí	AAB8578	KU134859	MCP-25476
*M. obtusidens*	Piracicaba (Tamanduá)^23^	Paraná	AAB8578	JN988985	LBPV-19849
*M. obtusidens*	Piracicaba (Tamanduá)^23^	Paraná	AAB8578	JN988984	LBPV-19850
*M. obtusidens*	Piracicaba (Tamanduá)^23^	Paraná	AAB8578	JN988983	LBPV-19852
*M. obtusidens*	Paranapanema (Canoas)^22^	Paraná	AAB8578	KM897227	^∗^
*M. obtusidens*	Paranapanema (Canoas)^22^	Paraná	AAB8578	KM897138	^∗^
*M. obtusidens*	Paranapanema (Canoas)^22^	Paraná	AAB8578	KM897434	^∗^
*M. obtusidens*	Cinzas (Bandeirantes)^25^	Paraná	AAB8578	KM897597	^∗^
*M. obtusidens*	Turvo (Icem)^21^	Paraná	AAB8578	KX925480	LISDEBE 6969
*M. obtusidens*	Turvo (Icem)^21^	Paraná	AAB8578	KX925481	–
*M. obtusidens*	Turvo (Icem)^21^	Paraná	AAB8578	KX925482	–
*M. obtusidens*	Turvo (Icem)^21^	Paraná	AAB8578	KX925483	–
*M. obtusidens*	Turvo (Icem)^21^	Paraná	AAB8578	KU134858	MZUSP – 113982
*M. obtusidens*	Turvo (Icem)^21^	Paraná	AAB8578	KF568987	–
*M. obtusidens*	Paraná (Porto Camargo)^24^	Paraná	AAB8578	KX925484	–
*M. obtusidens*	Paraná (Porto Camargo)^24^	Paraná	AAB8578	KX925485	–
*M. obtusidens*	Ibicui (BR 472)^25^	Uruguay	AAB8578	KU134860	MCP-28917
*M. piavussu*	Piracicaba (Tamanduá)^23^	Paraná	AAB8569	JN989005	LBPV-15587^∗^
*M. piavussu*	Piracicaba (Tamanduá)^23^	Paraná	AAB8569	JN989004	LBPV-19851^∗^
*M. piavussu*	Piracicaba (Tamanduá)^23^	Paraná	AAB8569	JN989003	LBPV-19854^∗^
*M. piavussu*	Paranapanema (Canoas)^22^	Paraná	AAB8569	KM897529	^∗^
*M. piavussu*	Paranapanema (Canoas)^22^	Paraná	AAB8569	KM897489	^∗^
*M. piavussu*	Paranapanema (Canoas)^22^	Paraná	AAB8569	KM897621	^∗^
*M. piavussu*	Cinzas (Bandeirantes)^25^	Paraná	AAB8569	KM897419	^∗^
*M. piavussu*	Paranapanema (Canoas II)^31^	Paraná	AAB8569	KM897506	^∗^
*M. piavussu*	Paranapanema (Canoas II)^31^	Paraná	AAB8569	KM897347	^∗^
*M. piavussu*	Paranapanema (Canoas II)^31^	Paraná	AAB8569	KM897192	^∗^
*M. piavussu*	Turvo (Icem)^21^	Paraná	AAB8569	KF568991	MZUSP – 113981
*M. piavussu*	Turvo (Icem)^21^	Paraná	AAB8569	KX925486	LISDEBE 6968
*M. piavussu*	Turvo (Icem)^21^	Paraná	AAB8569	KX925487	LISDEBE 6970
*M. piavussu*	Turvo (Icem)^21^	Paraná	AAB8569	KX925488	LISDEBE 6970
*M. piavussu*	Turvo (Icem)^21^	Paraná	AAB8569	KX925489	–
*M. piavussu*	Turvo (Icem)^21^	Paraná	AAB8569	KX925490	–
*M. piavussu*	Turvo (Icem)^21^	Paraná	AAB8569	KX925491	–
*M. piavussu*	Paraná (Porto Camargo)^24^	Paraná	AAB8569	KX925492	–
*M. piavussu*	Paraná (Pauliceia)^30^	Paraná	AAB8569	KX925493	–
*M.* cf. *piavussu* lower Paraná	Paraná (Rosario)^32^	Paraná	AAB8569	KU288864	^∗^
*M.* cf. *piavussu* lower Paraná	Paraná (Rosario)^32^	Paraná	AAB8569	KU288865	^∗^
*M.* cf. *piavussu* lower Paraná	Paraná (Rosario)^32^	Paraná	AAB8569	KU288866	^∗^
*M.* cf. *piavussu* lower Paraná	Paraná (Rosario)^32^	Paraná	AAB8569	KU289030	^∗^
*M. reinhardti*	Pandeiros^14^	São Francisco	AAD1729	HM906025	^∗^
*M. reinhardti*	Curimataí^17^	São Francisco	AAD1729	HM405147	MCP – 44776^∗^
*M. reinhardti*	Curimataí^17^	São Francisco	AAD1729	HM906026	^∗^
*M. reinhardti*	Urucuia (Urucuia)^13^	São Francisco	AAD1729	HM906027	^∗^
*M. reinhardti*	Urucuia (Urucuia)^13^	São Francisco	AAD1729	HM906028	^∗^
*M. reinhardti*	São Francisco (Três Marias)^16^	São Francisco	AAD1729	KX925494	–
*M. reinhardti*	Urucuia (Urucuia)^13^	São Francisco	AAD1729	KX925495	–
*M. reinhardti*	Urucuia (Urucuia)^13^	São Francisco	AAD1729	KX925496	–
*M. reinhardti*	Curimataí^17^	São Francisco	AAD1729	KX925497	MCP - 44770
*M. trifasciatus*	Madeira^6^	Amazonas	ACL3073	KU134864	UFRO-I 4902
*M. trifasciatus*	Ucayali (Pucallpa)^5^	Amazonas	ACL3073	KU134865	MUSM - 47351
*M. trifasciatus*	Ucayali (Pucallpa)^5^	Amazonas	ACL3073	KX925505	MUSM - 47351
*M. trifasciatus*	Amazonas (Belen)^3^	Amazonas	ACL3073	KX925506	MUSM - 47364
*M. trifasciatus*	Lago Catalão (Manaus)^4^	Amazonas	ACL3073	KX925507	INPA 11641
*M. reinhardti*	Urucuia (Urucuia)^13^	São Francisco	AAD1729	HM906028	^∗^
*M. reinhardti*	São Francisco (Três Marias)^16^	São Francisco	AAD1729	KX925494	–
*M. reinhardti*	Urucuia (Urucuia)^13^	São Francisco	AAD1729	KX925495	–
*M. reinhardti*	Urucuia (Urucuia)^13^	São Francisco	AAD1729	KX925496	–
*M. reinhardti*	Curimataí^17^	São Francisco	AAD1729	KX925497	MCP - 44770
*M. trifasciatus*	Madeira^6^	Amazonas	ACL3073	KU134864	UFRO-I 4902
*M. trifasciatus*	Ucayali (Pucallpa)^5^	Amazonas	ACL3073	KU134865	MUSM - 47351
*M. trifasciatus*	Ucayali (Pucallpa)^5^	Amazonas	ACL3073	KX925505	MUSM - 47351
*M. trifasciatus*	Amazonas (Belen)^3^	Amazonas	ACL3073	KX925506	MUSM - 47364
*M. trifasciatus*	Lago Catalão (Manaus)^4^	Amazonas	ACL3073	KX925507	INPA 11641

*^∗^Obtained from BOLD.*

### DNA Extraction, Amplification, and Sequencing

Total DNA was extracted from tissues (fins, muscle, or liver) by the standard phenol–chloroform method (Sambrook et al., 1989). A fragment of Cytochrome Oxidase subunit I (COI; 698 bp) was amplified via polymerase chain reaction (PCR) using primers AnosCOIF and AnosCOIR (Ramirez and Galetti, 2015). PCR products were sequenced for both strands using an ABI 3730xl (Applied Biosystems, Waltham, MA, United States) automatic sequencer. Contigs were assembled and edited using BioEdit (Hall, 1999). All sequences were evaluated manually, deleting regions of low quality. All sequences were verified to represent the COI gene and were checked for indels and stop codons. GenBank (Benson et al., 2017) accession numbers are given in **Table 1**. All information about specimen, sequences, and electropherograms were deposited in a data set of The Barcode of Life Database platform (BOLD) with code DS-MGLEP.

### DNA Barcode Analysis

The general mixed Yule coalescent (GMYC) model (Pons et al., 2006) with a single threshold, implemented in the *splits* packages in the R 3.3.3 statistical software (R Core Team, 2017), was used to infer MOTUs. For the GMYC input, an ultrametric tree was generated using Beast 2.4.3 (Bouckaert et al., 2014), with a lognormal relaxed clock, a birth and death model, and a GTR+G substitution model, chosen using jModeltest 2 (Darriba et al., 2012), using 50 million MCMC generations and a burn-in of 10%. Poisson tree processes (PTP) model (Zhang et al., 2013) was used for MOTUs delimitation through the bPTP server^1^, using default values. The bPTP server includes a Bayesian implementation of the PTP model and the original maximum likelihood PTP. For the PTP input, a tree was generated using Beast 2.4.6 (Bouckaert et al., 2014), with a strict clock, a birth and death model, and the GTR+G substitution model, using 50 million MCMC generations and a burn-in of 10%.

Additionally, two cluster algorithms were used, the Barcode Index Number System (BIN) (Ratnasingham and Hebert, 2013) and Automatic Barcode Gap Discovery (ABGD) (Puillandre et al., 2012). The BIN was automatically determined in the BOLD Workbench, while the ABGD was performed using Kimura-2-parameter (K2P) distance and default values through the web interface^2^.

COI intraspecific and interspecific genetic distances were estimated using the K2P model implemented in Mega 6.0 (Tamura et al., 2013). These values were used to calculate the mean, minimum, and maximum values for intra- and inter-MOTU distances, and intra- and interspecific distances (nominal species). A genetic distance neighbor-joining (NJ) tree analysis was performed based on the K2P substitution model in Mega 6.0 (Tamura et al., 2013).

## Results

**FIGURE 3:**
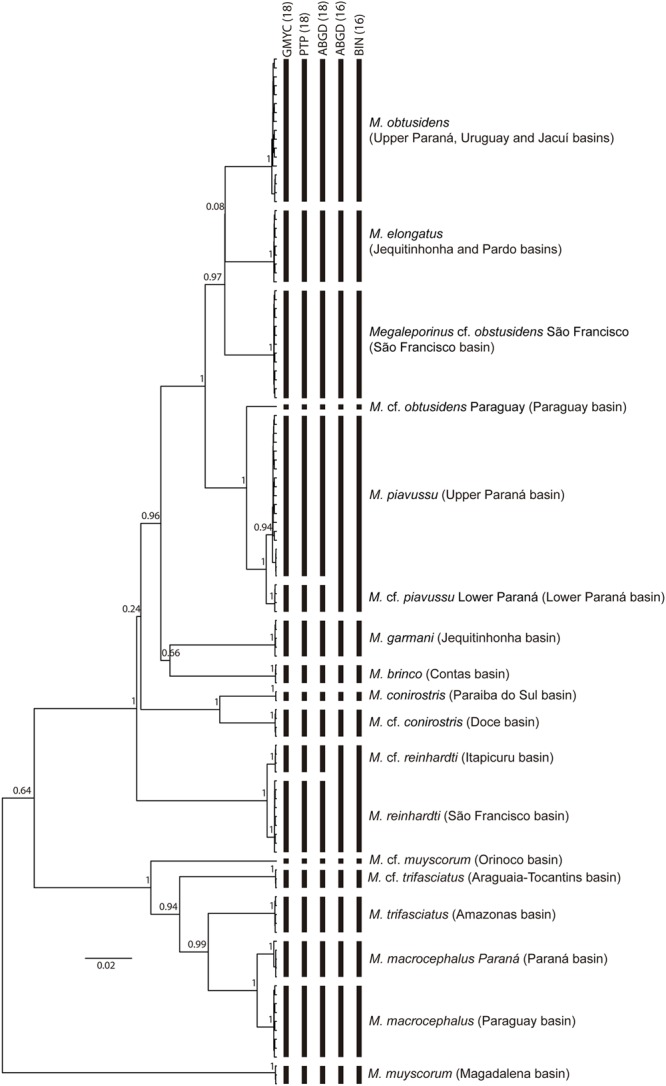
Bayesian tree showing the clustering of the MOTUs obtained by the species delimitation analyses.

The alignment of COI sequences resulted in 600 characters with 158 parsimony informative sites (included in the Supplementary Material). The GMYC analysis resulted in 18 MOTUs (Confidence interval: 16–18) (**Table 2**). The GMYC model was preferred over the null model (likelihood ratio = 73.49, *P* < 0.0001), indicating that GMYC results are reliable. The PTP analyses (maximum likelihood and Bayesian implementation) resulted in the same 18 MOTUs obtained in GMYC. The ABGD analysis found six partitions with 27 (*P* = 0.001) to 16 groups (*P* = 0.01), including a partition with the same 18 MOTUs (*P* = 0.005) obtained in the GMYC and PTP analyses. The BOLD system determined 16 BINs (**Table 2**), showing discordance with our MOTUs in only two BINs, AAB8569 [*M. piavussu* (Britski et al., 2012) and *M.* cf. *piavussu* lower Paraná] and AAD1729 [*M. reinhardti* (Lütken, 1875) and *M.* cf. *reinhardti*]. The clustering of the MOTUs obtained by the analyses is shown in **Figure 3**.

**Table 2:** Genetic K2P distances of *Megaleporinus* species.

	Mean intra-	Maximum intra-	NN	Distance to NN
**MOTUs**
*Megaleporinus brinco*	0	0	*M. obtusidens*	6.78
*M. conirostris*	0	0	*M. cf. conirostris*	3.99
*M. cf. conirostris*	0	0	*M. conirostris*	3.99
*M. elongatus*	0.04	0.17	*M. cf. obtusidens* São Francisco	2.74
*M. garmani*	0	0	*M. obtusidens*	7.68
*M. macrocephalus*	0	0	*M. macrocephalus* Paraná	1.86
*M. macrocephalus* Paraná	0	0	*M. macrocephalus*	1.86
*M. muyscorum*	0	0	*M. reinhardti*	11.6
*M. cf. muyscorum*	–	–	*M. trifasciatus*	7.48
*M. obtusidens*	0.14	0.5	*M. cf. obtusidens* São Francisco	2.84
*M. cf. obtusidens* Paraguay	–	–	*M. piavussu* Lower Paranáa	2.9
*M. cf. obtusidens* São Francisco	0	0	*M. elongatus*	2.74
*M. piavussu*	0.06	0.17	*M. piavussu* Lower Paraná	0.67
*M. piavussu* Lower Paraná	0.08	0.17	*M. piavussu*	0.67
*M. reinhardti*	0	0	*M. cf. reinhardti*	0.67
*M. cf. reinhardti*	0	0	*M. reinhardti*	0.67
*M. trifasciatus*	0	0	*M. macrocephalus*	4.52
*M. cf. trifasciatus*	0	0	*M. trifasciatus*	6.33
**Nominal**
*Megaleporinus brinco*	0	0	*M. obtusidens*	6.78
*M. conirostris*	2.13	3.99	*M. obtusidens*	5.6
*M. elongatus*	0.04	0.17	*M. obtusidens*	2.74
*M. garmani*	0	0	*M. obtusidens*	7.68
*M. macrocephalus*	0.86	1.86	*M. trifasciatus*	4.52
*M. muyscorum*	7.66	15.31	*M. trifasciatus*	7.48
*M. obtusidens*	1.94	6.72	*M. elongatus*	2.74
*M. piavussu*	0.26	1.01	*M. obtusidens*	2.9
*M. reinhardti*	0.31	0.7	*M. conirostris*	6.14
*M. trifasciatus*	3.39	6.33	*M. macrocephalus*	4.52

*The mean and the maximum of intra-group distances, the nearest neighbor (NN), and the minimum distance to the NN for MOTUs (ABGD, GMYC, and PTP) and Nominal species.*

Only *Megaleporinus brinco* (Birindelli and Britski, 2013), *Megaleporinus garmani* (Borodin, 1929), and *Megaleporinus elongatus* (Valenciennes, 1850) showed correspondence between nominal species and MOTUs. Within six nominal species, a subdivision in two MOTUs was found, while *Megaleporinus obtusidens* (Valenciennes, 1837) was divided in three MOTUs (**Table 2**).

The mean of intra-MOTU and maximum intra-MOTU distances, the nearest neighbor (NN), and the minimum distance to the NN are shown in **Table 2**, for both GMYC MOTUs and nominal species.

The overall mean of intra-MOTU distances was 0.03%, the maximum intra-MOTU distance was 0.5% (*M. obtusidens*), and the mean of inter-MOTU distances was 9.19%. The lowest and highest values of inter-MOTU distances were 0.67 and 15.31%, respectively. Considering these values, there is a barcode gap that allowed identifying successfully all MOTUs using COI distance. In contrast, when only the nominal species were considered, the maximum intraspecific distance increased to 15.31% [*M. muyscorum* (Steindachner, 1900)], and, in addition, no barcode gap was found.

## Discussion

Our hypothesis that some of the nominal species represent species complexes separated in different basins could not be rejected by DNA barcoding analysis, revealing taxonomic uncertainties and a hidden diversity within this recently described genus. The DNA barcode analyses identified 16 (ABGD and BIN) to 18 (ABGD, GMYC, and PTP) different MOTUs (**Figure 3**), with two new MOTUs (*M*. *macrocephalus* Paraná and *M.* cf. *piavussu* lower Paraná) not analyzed by Ramirez et al. (2017). This high number of MOTUs contrasts with the 10 nominal species recognized in the genus thus far, showing several potential target for cryptic species to be described, reinforcing the general idea that there is still a lot of undocumented diversity within the neotropical ichthyofauna (Reis et al., 2016). The difference between the number of MOTUs detected is due to the lower genetic distance value (0.67%) between two pairs of MOTUs: *M. reinhardti* and *M.* cf. *reinhardti*, separating the genetic lineages from São Francisco and Itapicuru, respectively, and between *M. piavussu* and *M.* cf. *piavussu* lower Paraná. These lower genetic distance values are likely due to a recent divergence between these MOTUs [<0.5 Ma for *M. reinhardti* and *M.* cf. *reinhardti* according to Ramirez et al. (2017)]. Of note, besides presenting an allopatric distribution, these MOTUs were also recovered by the monophyly criterion (**Figure 3**). MOTUs with recent origin have less time to accumulate genetic differences than species with ancient origin, hindering their identification. Despite this low genetic distance, the species delimitation methods could delimit these MOTUS, especially those based on phylogenetic trees (GMYC and PTP).

A key aspect implicit in the DNA barcoding analysis is the genetic distance threshold used to define MOTUs. COI distances of 1% (Hubert et al., 2008) to 2% (Pereira et al., 2013) have been claimed as threshold to fish DNA barcode analysis. However, such values were derived from comparative analyses among phylogenetically diverse groups. For instance, 2% was used to characterize DNA barcoding of a fish community of a given river (Pereira et al., 2013). However, when the DNA barcoding analyses have focused within a group of species closely related (e.g., a genus), lower threshold values have been reported (Carvalho et al., 2011; Pereira et al., 2011, 2013; Ramirez and Galetti, 2015). Particularly in Anostomidae, a lower threshold of 0.92% was reported to distinguish MOTUs within the genus *Laemolyta* (Ramirez and Galetti, 2015). Although most of the values obtained herein were above 2% (13 out of 18 MOTUs, **Table 2**), a maximum threshold of 0.67% for *Megaleporinus* was detected between the MOTUs obtained. It reinforces that lower genetic distance values might be obtained when intra-genus MOTUs are analyzed, mainly between recent divergent lineages.

Five nominal species, *M. conirostris* (Steindachner, 1875), *M. macrocephalus* (Garavello and Britski, 1988), *M. muyscorum, M. obtusidens*, and *M. trifasciatus* (Steindachner, 1876), showed high COI distance values (> 1.8%, **Table 2**) between individuals from different basins, indicating a scenario of potential allopatric speciation within these species.

In contrast to previous results (Avelino et al., 2015), evidence of local differentiation was not found here and all cryptic diversity correspond to inter-basin differentiation. Analyzing only two samples of *M. reinhardti* from the Três Marias (MG, Brazil) region (São Francisco basin), Avelino et al. (2015) reported an intraspecific distance of 3.8% between them, suggesting a local differentiation. Here we analyzed nine individuals, representing four different localities, including Três Marias region, and we found no genetic distance (0%) among them. Mitochondrial pseudogenes, sequencing errors, or misidentification could explain such discrepancies, and it would be more cautious to consider *M*. *reinhardti* from São Francisco as a single MOTU, as recovered here.

Similar discordance is observed for *M. piavussu* (upper Paraná). Avelino et al. (2015) included four samples from a single locality and reported a mean intraspecific distance of 2.8%. Our present data set for this species included 18 individuals obtained from six localities and showed a lower maximum intraspecific distance of 0.17%. It is strongly suggested that *M. piavussu* is also a single MOTU.

Incongruences were also observed within the nominal *M. obtusidens*. While four groups (A–D), showing 0.7–4.1% mean intraspecific distances, were previously reported (Avelino et al., 2015), we found three MOTUs showing 0–0.5% COI distances. The group D mentioned as part of *M. obtusidens* by Avelino et al. (2015), which included individuals caught downstream the Itaipú dam (Paraná basin), was recovered here as a sister group of *M. piavussu*, and was named *M.* cf. *piavussu* lower Paraná (**Figure 3**).

One particular aspect was highlighted in our results. Several individuals clustered in the *M. macrocephalus* clade were caught in different hydrographic basins, as Doce, São Francisco, Tocantins, and Paraná, outside of its original distribution in the Paraguay basin likely due to aquaculture releasing. Similar findings had already been described in the São Francisco basin (Carvalho et al., 2011). This species is a commercial important fish being extensively farmed throughout the Brazilian territory, and accidental or intentional releasing can occur (e.g., Langeani et al., 2007; Vieira, 2010). In such case, the use of DNA barcoding provides a rapid and accurate identification of this species and can be used in management and monitoring potential ecosystem disturbance caused by an invasive species.

In summary, the use of DNA barcoding points at the need for a taxonomic revision of this genus. A search for morphological traits able to support a taxonomic delimitation could be facilitated whether the MOTUs identified here are considered. A morphological trait showing a range of variation when searched within a given nominal species perhaps could be more informative if studied in each MOTU separately. In such case, our results would give an important contribution for the taxonomy of *Megaleporinus* facilitating the search for decisive taxonomic characters. This is the most complete analysis of DNA barcode in this recently described genus, and considering the economic value of this group, a precise species identification is quite desirable and fundamental for conservation of the whole biodiversity of this genus.

## Author Contributions

JR and PG designed the research. JR, DC, PA, PV, HO, MC-A, and JR-P collected data. JR performed the analyses. All authors contributed to the writing of the manuscript.

## Conflict of Interest Statement

The authors declare that the research was conducted in the absence of any commercial or financial relationships that could be construed as a potential conflict of interest.

## Footnotes

1 http://species.h-its.org/ptp/

2 http://wwwabi.snv.jussieu.fr/public/abgd/abgdweb.html
